# “It was actually my family and friends who noticed that my voice changed”: An interview study on the experiences of transgender and non-binary youth and young adults three years into medical treatment

**DOI:** 10.1080/26895269.2025.2478498

**Published:** 2025-03-15

**Authors:** Reidar Schei Jessen, Anne Wæhre, Linda W. David, Line Indrevoll Stänicke, Erik Stänicke

**Affiliations:** a Department of Psychology, University of Oslo, Oslo, Norway; b Division of Pediatric & Adolescent Medicine, Oslo University Hospital, Oslo, Norway

**Keywords:** Gender-affirming care, transgender and non-binary, youth and young adults

## Abstract

**Objective:**

As the number of transgender and non-binary (TGN) youth and young adults referred for gender-affirming medical treatment increases, there is a need to better understand how these individuals experience the interventions. The aim of the present study is to expand our understanding of how TGN youth and young adults perceive bodily changes following medical treatment as part of gender-affirming care.

**Method:**

Ten life-mode interviews exploring individuals’ everyday experiences and the meanings they attach to them were conducted with TGN youth and young adults in Norway, assigned female at birth, aged 17–22, three years after they initiated gender-affirming medical treatment to align their bodies with their gender identities. The data were analyzed using thematic analysis.

**Results:**

Based on the analysis of the semi-structured interviews, we developed three major themes and six sub-themes: (1) *A pragmatic approach to making decisions regarding medical treatment* indicates that the participants are continuously weighing the risks and benefits, together with a gradual, step-by-step introduction of medical treatment. (2) *Embodying medical treatment* indicates that bodily changes are tangible but, over time, anchor a sense of masculinity and a sense of self as male. (3) *Grounding medical treatment in everyday life* indicates that bodily changes help connect the inside with the outside, with family and friends serving as sources of support and mediators of meaning, highlighting the psychosocial aspects of gender-affirming care.

**Conclusion:**

The psychosocial aspects related to medical treatment in gender-affirming care are important vantage points for exploring the social meaning of the body and bodily changes. We therefore suggest that psychosocial aspects of gender-affirming care should be attended to and integrated alongside medical treatment.

## Introduction

For some transgender and non-binary (TGN) individuals, a lack of bodily alignment with their gender identity causes distress, referred to as gender incongruence in ICD-11 (World Health Organization, 2019) and gender dysphoria in DSM-5 (American Psychiatric Association, 2013). This distress often leads TGN individuals to seek various treatments, such as hormone therapy, surgeries, and counseling, which are collectively referred to as gender-affirming care, to achieve bodily alignment with their gender identity (Coleman et al., 2022).

A systematic review of qualitative research on the subjective experiences of gender among TGN youth and young adults reported that gender incongruence is a social transaction between factors such as the body, sexual impulses, and relationships in which each person participates (Jessen et al., 2021a). While correlational outcome research on medical treatment in gender-affirming care, which uses questionnaires to assess psychiatric symptoms and quality of life measures, provides valuable insights, it should be complemented with qualitative studies of lived experiences. This combined approach can help explore the potential mechanisms through which gender-affirming medical interventions improve overall quality of life and psychosocial functioning (Chen et al., 2023; Coleman et al., 2022). However, questions remain unanswered regarding the timing of medical treatment and how it relates to other developmental milestones in adolescence and young adulthood, such as identity exploration, sexuality, and the increasing demands from the adult world (de Vries, 2020; Gridley et al., 2016).

Even though clinical treatment guidelines and follow-up studies recommend psychosocial and family support alongside medical treatment in gender-affirming care (Coleman et al., 2022; Katz-Wise et al., 2022), less is known about how this should be integrated on an individual level (Jessen et al., 2021a). The disconnect between the body and an individual’s internal sense of gender is mentioned by young people themselves as a key reason for seeking medical treatment (Katz-Wise et al., 2022). The decision-making process leading to the initiation of medical interventions typically begins with the young people exploring their needs independently, followed by discussions with parents and care providers (Clark et al., 2020). Some young people report having already made decisions regarding medical treatment, while others describe a process of reflection after being referred to a gender-affirming clinic (Clark et al., 2020; Sansfaçon et al., 2019). Thus, youth and young adults’ gender identities continue to evolve during the course of gender-affirming care and through the physical changes following medical treatment (Clark et al., 2020; Sansfaçon et al., 2019).

A common denominator across the qualitative literature on TGN youth and young adults and their experiences in relation to medical treatment is the important role that family, friends, school, and others that form part of the social environment play (Katz-Wise et al., 2022; Kearns et al., 2021; Riggs et al., 2020;). Furthermore, TGN youth and young adults highlight that a valued aspect of their relationship with parents is that their parents continue treating them as the same person while also allowing them to change their understanding of themselves in terms of gender (Riggs et al., 2020). Parents can also be important advocates for their child by translating their needs in relation to for example hospital or school (Kearns et al., 2021; Riggs et al., 2020;). Moreover, some TGN young people refer to parental denial of hormonal treatment as “blockers to a sense of self” (Riggs et al., 2020). Thus, the process of making decisions regarding medical treatment as part of gender-affirming care can be interpreted as a question of whether parents and providers believe in the young person’s self-understanding (Kearns et al., 2021; Riggs et al., 2020). Furthermore, physical changes after medical interventions helped many young people to be perceived as their gender identity, enabling further exploration of gender through feedback from others (Sansfaçon et al., 2020). Moreover, even in the absence of perceived changes in the body following medical treatment, accessing medical treatment was described as an improvement in their well-being because it was perceived as a step in the right direction (Sansfaçon et al., 2019). In addition, it can be challenging to put gender-related thoughts and feelings into words, making the communication process with healthcare personnel both important and potentially sensitive (Turban et al., 2017).

### The present study

The overarching aim of the present study is to expand our understanding of how TGN youth and young adults in Norway experience bodily changes following medical treatment as part of gender-affirming care. Having previously identified that gender identity is a complex social and bodily phenomenon that must be continuously negotiated (Jessen et al., 2021b), we now explore experiences with treatment three years after initiating medical treatment in gender-affirming care. More precisely, the study was guided by the following research questions:

1. What are the lived experiences of TGN youth and young adults concerning bodily changes following medical treatment, and how do these changes impact their sense of identity and well-being in everyday life?
2. How do they make decisions regarding medical treatment, and what types of social support in everyday life have been important?

## Method

### Qualitative approach and research paradigm

To explore lived experiences related to medical treatment in gender-affirming care, the study was guided by a phenomenological perspective as a theoretical framework to uncover the essence of the experiential aspects of individuals’ being in the world that are often taken for granted (Dahlberg, 2006). Furthermore, we chose a qualitative semi-structured interview approach because it is a suitable strategy for exploring people’s meaning-making and the processes behind various treatment outcomes (McLeod, 2014).

### Context and study setting

The study was designed by a team comprising of four researchers with experience as clinical psychologists (the first, third, fourth and last authors) and a child and adolescent psychiatrist (the second author) in collaboration with three user organizations. During the time of the study, the second and third authors worked at the Norwegian National Center for Gender Incongruence in clinical positions. The clinic-based setting might have influenced participants in the sense that they were prompted to focus on topics that pertain to a clinical assessment instead of a holistic focus. To counter this and promote a contextual approach, the interviews were conducted outside of the clinic, and participants were repeatedly reminded throughout the interview that non-treatment experiences were also relevant.

### Researcher characteristics and reflexivity

According to Levitt et al. (2018), maintaining awareness of the researcher’s influence on the research process and findings is also an important measure to enhance thrustworthiness. In line with the suggestions by May and Perry (2013), we strived to continuously discuss the strengths and weaknesses of our methodological approach, including reflecting on our own pre-understanding of transgender identity and investments in certain models of care within the gender-affirming approach. This was especially important as none of the authors have personal experience as transgender individuals. Two of the authors are cisgender men, and three are cisgender women. Based on feedback from user representatives during the first round of interviews three years ago, we were aware that, as clinicians, we might have a bias toward focusing on suffering at the expense of highlighting resilience and gender euphoria. To counter this tendency, we systematically focused on experiences regarding resilience throughout the analysis. The choice of phenomenology as a guiding theory and the focus on first-hand experiences during the early phases of the analysis was also an attempt to stay as close as possible to lived experiences of young transgender people. Furthermore, the first author has been politically active in LGBTIQ+ work and maintains a close connection with various user-led initiatives to continuously discuss the potential implications of the research findings. The first author has previously published an autobiographical study on strategies for how a cisgender researcher can maintain a reflexive stance when conducting research with transgender people (Jessen, 2024).

### Participants and sampling strategy

The sample consisted of ten participants assigned female at birth, aged 17 to 22, who initiated gender-affirming care three years ago at the Norwegian National Center for Gender Incongruence. During their treatment at this clinic three years ago, they participated in an interview conducted by the first author about their subjective experiences of gender and their expectations before starting medical treatment. We strived to ensure a diverse background among the participants in terms of gender identity, birth-assigned sex, and age during recruitment. Before the first interview, participants received a written request for participation and were given the option to contact the first author if they wanted to take part. They also agreed to be contacted again. Three years later, 13 of the 16 original participants consented to participate when approached by the first author. One participant from the initial sample declined to participate again, while two did not respond to the requests. Among the 13 who consented to participate in the follow-up interview, two never initiated treatment, and one discontinued after just a few months due to medical complications. Thus, the ten participants in the present study represent the experiences of those who ultimately pursued medical treatment.

Common to all ten participants is that none of them suffer from psychosis or severe suicidal behavior. The characteristics of the present follow-up sample are presented in Table 1. All participants have pursued hormonal treatment (testosterone); five have pursued chest surgery (mastectomy), and one has had a hysterectomy. Five of the participants had been on hormonal treatment for three years, four for two years, and one for one year. Two participants had undergone chest surgery three years ago, one participant two years ago, and two participants within the last year. Three participants reported a history of questioning their gender identity in early childhood, while the remaining seven reported they began questioning their gender identity after the onset of puberty. Additionally, two participants were people of color, while eight were Caucasian. However, when presenting the results, we concealed this information to protect participant anonymity, considering the small population of TGN youth and young adults assigned female at birth seeking gender-affirming care. The participants were not remunerated.

**Table 1.** Sample characteristics (*n* = 10).

Age range: 17–22.
Medical treatment
Testosterone treatment (initiated between 2 and 4 years ago): *n* = 10
Chest surgery: *n* = 5
Hysterectomy (removal of uterus): *n* = 1
Education and employment status
High school: *n* = 3
Student: *n* = 2
Working full-time: *n* = 4
Gap year: *n* = 1
Home context
Living with parents: *n* = 4
Living with friends: *n* = 5
Living with partner: *n* = 1
Gender
Female sex assigned at birth: *n* = 10
Binary gender identity: *n* = 9
Non-binary gender identity: *n* = 1
Debut of gender distress in childhood: *n* = 3
Debut of gender distress after puberty: *n* = 7

### Ethical considerations

The current study was approved by the Norwegian Regional Committees for Medical and Health Research Ethics (REK sør-øst) (2018/1088). The first author is a clinical psychologist, and measures were taken before, during, and after the interviews to ensure that the participants’ mental health and well-being were attended to. Additionally, participants had the option to contact their clinical contact at the Norwegian National Center for Gender Incongruence if the interviews elicited any traumatic or powerful emotional memories. Furthermore, several measures were implemented to obtain consent. Firstly, at several points during the interview, the interviewer ensured that participants could choose to discontinue. Secondly, participants were informed that their gender-affirming care at the Norwegian National Center for Gender Incongruence would not be contingent upon research participation. These measures were important to ensure the participants’ autonomy and integrity, especially since they belonged to a vulnerable group as minorities and patients receiving healthcare, and considering that the interviewer was a clinical psychologist (Levitt et al., 2018).

### Data collection methods

The young people participated in personal interviews that followed a semi-structured approach. The shortest interview lasted 77 min, while the longest was 116 min, with an average length of one hour and 40 min. All interviews were conducted by the first author. In the first part of the interview, participants were encouraged to describe what had happened in their lives since the initial interview, focusing on both medical treatment and important life events such as school, university, job, friends, and intimate relationships. The focus included both challenges and improvements in their lives. In the second part, the life-mode interview approach was employed, asking participants to recount the events of the day preceding the interview, from waking up to going to bed (Haavind, 2019). This approach was chosen because it effectively elicits detailed and contextual descriptions of lived experiences, emphasizing the affective, material, and relational aspects of these events (Haavind, 2019). In this case, the interviewer asked for concrete experiences related to treatment topics, such as the effects and expectations of medical treatment, decision-making, and communication with healthcare personnel and family.

Before the study was initiated, the first author conducted a pilot interview to test the validity and usefulness of the interview guide. The first author’s experiences from the pilot interview were then discussed with the coauthors, and the guide was lightly revised to better capture treatment-relevant experiences (Levitt et al., 2018). Additionally, the first author made field notes during the interviews, consisting of reflections and recurrent themes, which were discussed with the coauthors over the three-month period when the interviews took place. Six of the interviews were conducted in the participants’ local environments (mostly public libraries), one *via* Zoom because of long travel distances, and three at the first author’s university office.

### Data analysis

The interviews were transcribed by a research assistant and subsequently reviewed by the first author, who also conducted the interviews. To protect participants’ privacy, they were given pseudonyms different from those used in the publications reporting on the analysis of the first wave of interviews (Jessen et al., 2021b; Jessen et al., 2025).

To grasp the breadth of the data and capture nuances in treatment experiences, we followed the approach of reflexive thematic analysis (Braun & Clarke, 2019). As a first step, the first author read and reread the interviews and wrote a narrative for each participant, summarizing the biographical data and the most significant life changes over the past three years. This approach was intended to maintain a vertical focus on individual complexity before analyzing horizontally across cases, aligning with the first phases of thematic analysis: (1) Familiarizing yourself with your data and (2) Generating initial codes (Braun & Clarke, 2019). The first author then developed 15 codes based on the initial in-depth reading of the interviews. The coauthors read summaries of the codes with illustrative quotes and discussed tentative themes across participants, a process corresponding to the third phase of thematic analysis: (3) Searching for themes. The first author translated the quotes, which were then revised by the coauthors. After all the authors had reviewed the initial 15 tentative codes, the first author suggested a revised set of six sub-themes and three major themes to form the basis of the results. This process corresponds to the fourth phase of thematic analysis: (4) Reviewing themes. Subsequently, the first author refined the specifics of the themes, performed a detailed analysis, and composed a draft of the results section, corresponding to the fifth and sixth steps of thematic analysis: (5) Defining and naming themes and (6) Producing the report (Braun & Clarke, 2019).

### Techniques to enhance trustworthiness

Several steps were taken to enhance the trustworthiness and methodological integrity of the study (Levitt et al., 2018). To maintain fidelity to the subject matter, we followed Watts (2014) recommendations for the skillful application of qualitative methods. This approach involved distinguishing between first-person and third-person perspectives during the analytic process and while writing the results section, to achieve the greatest possible closeness to the participants’ experiences. The first-person perspective implies an attempt to discuss the results without introducing external knowledge, while the third-person perspective allows for theoretical analysis of the data (Watts, 2014). In line with a phenomenological perspective (Dahlberg, 2006), we therefore tried to maintain a close focus on the participants’ experiences during the first two steps of the thematic analysis, before carefully beginning to introduce theoretical perspectives to enable an analysis across cases in the subsequent steps (Braun & Clarke, 2019).

## Results

In the following sections, we describe the three major themes that emerged in the interviews when participants talked about their experiences with treatment. The first major theme focuses on participants’ decision-making related to medical treatment, specifically the processes leading up to initiating bodily changes. The second major theme concentrates more specifically on how participants have experienced these bodily changes following medical treatment. The third major theme addresses how these bodily experiences are a social transaction with the external world, grounded in everyday contexts and networks of social support. In real life, these processes are overlapping and dynamic, demonstrating how socially embedded medical treatment is in gender-affirming care. However, we present the processes separately to gain a better understanding of how they unfold. The themes represent patterns across the sample, with quotes used to illustrate the various major themes and sub-themes.

### Major theme 1: a pragmatic approach to making decision regarding medical treatment

The participants described in detail how the implementation of medical treatment was a step-by-step process, carefully guided by weighing the benefits of various medical treatments against potential risks in their daily lives, typically through discussions with caregivers and family.

#### Sub-theme 1.1: weighing risks against benefits

The participants seem to weigh the risks associated with various medical treatments with the benefits in their everyday life. In general, they seem to opt for the less invasive interventions to alleviate gender distress. Emil, for example, would ideally opt for bottom surgery. However, on a day-to-day basis, he is able to manage his bodily distress, especially after he had breast surgery: “It’s okay [to urinate]. But I prefer to pee in the shower, because then I can stand upright. But otherwise [the genitals] aren’t really something that bothers me, I don’t think much about it, actually” (Emil, 21 years). Given the potential complications associated with bottom surgery, Emil further reflected:

The genitals need to be repaired again [after surgery], reconstructed, there are several infections due to the urinary tract and all that, and I just don’t want to, it’s more pain than I’m willing to endure. As long as I am comfortable with it [the genitals], it just sounds unnecessary to me. (Emil, 21 years)

However, later in the interview, Emil said that he will reconsider bottom surgery later on if the procedures are improved or his gender experiences changes. The perception of potential benefits and risks are highly subjective. Magnus, for example, acknowledged that his need to remove his uterus and ovaries was not because it changed the appearance of his body: “I can’t [feel the ovaries and uterus]. But I feel like it might make me even more comfortable in my own body, like I know then I have one less worry to think about” (Magnus, 19 years). When asked what lies beneath this concern, he further elaborated: “Because I don’t have a great need for biological children, I feel like they [the ovaries and uterus] are just in the way, in a sense” (Magnus, 19 years). Thus, it seems that the mere possibility of having children, as long as the ovaries and uterus are intact, causes distress for Magnus and outweighs the potential risks associated with the surgical intervention to remove them. Billie gave another example when he reflected on why he ended up removing his ovaries and uterus:

I was a bit back and forth on it in a way, because you do get sterilized and can’t have biological children in the same way. I could have frozen some eggs, but I chose not to do that either. And then there are many who say that having ovaries is not necessarily linked to being a woman. But, I mean, for me personally, it [ovaries and uterus] is generally considered a female organ. (Billie, 21 years)

Furthermore, all participants reported that health personnel and parents are important discussants when they are weighing risks and benefits:

I have talked a lot at [name of gender-affirming clinic] about it, we have talked a lot about the various surgeries, that there are a lot of complications with bottom surgery. I just spoke to the man who was going to do my breast surgery, and he said that it is a very standard operation when you do the top surgery, because it is very straight forward. (Jacob, 17 years)

According to many participants, care providers tend to communicate that medical treatment is a life-long treatment. Consequently, they must be certain about their before medical treatment is initiated: “They have to be sure that I can get it and stuff like that, because they said it wasn’t so easy that everyone could get it, [you] have to be sure” (Camilo, 22 years). The participants’ parents do also seem to stress the importance of being certain before decisions are made, and tend to focus on risks associated with medical treatment:

I think she [his mother] is just worried that there will be complications, she’s a bit like “is it going to be fine?” a bit nervous about it [upcoming breast surgery]. I understand that it’s a bit scary, but I can get a bit annoyed in a way, she might not fully understand how important it is to me, that I might rather want to hear “oh, so nice that it’s finally happening,” or “so nice that you got an update on when it’s going to happen and it’s going to be very exciting.” (Billie, 21 years)

Thus, it seems that the participants are guided by needs grounded in everyday life situations when weighing risks against benefits, while parents and caregivers tend to focus more on certainty and long-term effects from a more medical perspective.

#### Sub-theme 1.2: introducing medical treatment step-by-step

To gain time to weigh the risks against the benefits, many participants find it useful to aim for a step-by-step implementation of medical treatment. Jacob, for example, said it was helpful to begin with medication that stopped menstruation, before he proceeded on testosterone:

I started on some pills that made it so I didn’t get my period anymore, and that was a big relief in itself… because it [menstruation] wasn’t meant for me. And then it was like starting testosterone when I, I had to wait until I turned 16 first, I was well into my 15th year before we started talking about testosterone. (Jacob, 17 years)

For Anders, the most urgent medical intervention was to remove his breasts: “Bottom surgery isn’t as urgent as top surgery, because they [breasts] were much more of a hindrance in everyday life, I felt” (Anders, 20 years). Before he started on testosterone, Anders wanted to pursue bottom surgery, because he found it challenging to use his genitals. This has however changed over time, because testosterone caused clitoris to grow, which improved his sexual life: “The hormones actually make you grow a bit down there [clitoris], so in that sense, it’s easier… that [orgasm] is not a problem” (Anders, 20 years). Anders is therefore now more reluctant about genital surgery, and he feels that he has time to wait and see. Furthermore, the ongoing weighing of potential risks and benefits seems to be grounded in a pragmatic reasoning regarding how the participants can handle:

At least I don’t want [to undergo bottom surgery] with today’s technology, at least. And for now, I’m okay with everything down there [the genitals]. As long as it works, I’m fine with it. But if I were to have surgery, I would rather do it when the surgery is better. (Emil, 21 years)

To summarize, the participants appear to base their decision-making regarding medical treatment on concrete experiences of gender distress and a lack of alignment between body and gender identity in everyday life. Furthermore, and perhaps as a consequence of the significance of these tangible everyday situations, they seem to approach decision-making as a gradual and ongoing process. This observation leads us to question how they experience bodily changes, which is the second major theme.

### Major theme 2: embodying medical treatment – experiences with changing the body

While the first major theme explores how the participants make decisions regarding medical treatment, the second major theme examines how they experience the bodily changes once they have begun.

#### Sub-theme 2.1: bodily changes are tangible

At one level, the bodily changes are quite tangible and therefore easy to grasp. Typical tangible bodily changes are voice change, increased body hair, altered fat distribution and enlarged clitoris: “The first thing was actually the voice, and that I became much more sensitive to heat. Crazy sensitive to heat. Hair in places I didn’t know hair could grow” (Henrik, 22 years). Even if the bodily changes were positive, some participants described unexpected side effects: “When you get hot, you may start to feel itching on your neck and back, and it begins to both sting and itch, sort of” (Anders, 20 years). Over time, however, the tangible bodily changes have helped the participants feel better about themselves:

Slowly, I just became more and more confident in myself by seeing that “wow, now my body is changing in a very positive way.” At first, I noticed my voice changing, then it moved on to noticing that my body and, like, fat distribution and stuff just changed, and then hair came. It happened a bit gradually. (Filip, 19 years)

Thus, what began as small and almost unnoticeable bodily changes have gradually gained increasing significance.

#### Sub-theme 2.2: bodily changes are anchoring a sense of masculinity

Gradually and almost unnoticed, the various bodily changes have increasingly bridged the gap between the participants’ bodies and gender identities, to the extent that they experience less gender incongruence in everyday life. An important aspect of this process appears to be that these bodily changes have made the participants feel more comfortable as men:

Starting testosterone helped. I feel like before I was trying to prove something, but now I have, like… I don’t need to prove anything, I am who I am, regardless. So I’ve become more comfortable with myself and my own masculinity. (Emil, 21 years)

Furthermore, when gender distress emerges, it is easier for the participants to handle it, because they overall feel more masculine: “I feel that [gender incongruence] doesn’t really affect me so negatively after I started taking hormones, because my life is going much better, so I don’t really tend to think about it so much” (Filip, 19 years). In addition, it seems that bodily changes, especially testosterone, has changed the participants’ mindset in a more masculine way:

I mostly think about the external changes that happen when you start testosterone, but there are also a lot of changes in your mindset and emotions. Now, for example, I can get much angrier than before, I was never angry before. (Emil, 21 years)

Moreover, the participants feel that the bodily changes make them feel and experience the world in a similar way to cisgender men.^1^ Camilo, for example, said that he feels emotionally less volatile after starting on testosterone:

I changed my body and changed my feelings. And thoughts, that I don’t overthink and such things. I have the same mindset as boys do, because there are quite a few boys who don’t overthink at all. They let go of things more easily, they’re not as dramatic as girls are. (Camilo, 22 years)

Thus, is seems that Camilo interprets emotional changes as signs that he is becoming more like cisgender men. Jacob further elaborated: “A change occurs, your clitoris becomes much larger and more sensitive, you can clearly see the difference compared to a typical female body, you can see the change and you feel that it’s different” (Jacob, 17 years). Thus, even apparently tangible physical changes like the growth of the clitoris seem to require a reference to an external social norm, in this case the idea of a typical female body, to be interpreted as indicative of a more masculine body.

How do the participants learn about these social norms regarding gender? Other people seem to be important in the process of interpreting bodily changes:

Mom pointed and said, “Oh, you have broader shoulders,” which was very nice to hear. It was mostly other people who noticed things; I just gradually saw that “oh yeah, now it’s happening, now I became a bit more comfortable,” you know. (Filip, 19 years)

As Filip indicated, after his mother made him aware of the bodily changes, he gradually felt more comfortable in terms of gender himself. Some participants even experienced that other people noticed the bodily changes before they did themselves:

I didn’t notice myself, it was actually family and friends who noticed that my voice changed, like voice cracks all the time. It has calmed down a bit now, but it still happens. I also lost weight, and others noticed it before me. (Jacob, 17 years)

Thus, forming relationships with other people, especially close relationships, was important for the participants as a framework for interpreting their bodily changes:

I got together with my current partner around the same time I started taking testosterone, and it [testosterone] has made it easier to be comfortable in my body, because we [my partner and I] have different bodies, but we are both men and that’s okay. (Billie, 21 years)

Moreover, as exemplified by Billie, being intimate with his new boyfriend and being affirmed as a man were important factors in beginning to interpret his bodily changes as masculine, relative to social norms.

To summarize the second major theme, tangible bodily changes following medical treatment seem to translate into a heightened sense of masculinity and integration within the male community. However, this transition from tangible bodily changes to socially meaningful experiences does not happen automatically; it seems to occur within a social framework where family, friends, or intimate partners act as mediators of meaning. Thus, the beneficial effects of medical treatment in gender-affirming care are intricately entwined with psychosocial dynamics and guided by an internal logic that connects private experiences of the body with gender identity within broader social contexts. In the next and final major theme, we examine how this interpretative work is an ongoing process that unfolds in everyday life situations.

### Major theme 3: grounding medical treatment and bodily changes in everyday life

As indicated in the second major theme, an important aspect of what makes medical treatment useful in connecting the internal sense of self with a broader social context is how participants interpret bodily changes in their daily lives. In the third major theme, we look more closely at the social processes in which this work of meaning-making is embedded.

#### Sub-theme 3.1: medical treatment helps connect the inside with the outside

According to the participants, the aim of medical treatment in gender-affirming care is, at one level, quite straightforward: to bring the body more in accordance with gender identity and gender expression. In general, the participants feel that the medical treatment has made it easier for them to experience coherence between their gender identity and how they are perceived by others: “Medical treatment is for myself. I want my body to look like that, and I want to sound like that, but also, it makes society and the world in general see me more as a boy too” (William, 17 years). In this way, William illustrates the dual purpose of gender-affirming care: to alter the body so that it becomes more comfortable for the participants themselves, and to make them intelligible as men in the eyes of others. However, as another participant emphasized, it can sometimes be challenging to balance the desire to be recognized as a man by others with the need to feel authentic and true to oneself:

I would like to be seen as a man, and it would have been easier for me to be recognized as a man by society if I simply cut my hair short, dyed it brown, and wore a regular t-shirt every day. Some days I wish I could have done that. But then I would also like to maintain my identity (Billie, 21 years)

Thus, as indicated by Billie, connecting one’s internal sense of self with an external gender expression seems to be an ongoing negotiation with the external world. However, as Jacob exemplified, it was necessary to start with some form of medical treatment to explore a new sense of self in terms of gender:

Medical treatment played a much larger part in my life two, three years ago, because then I had many more issues with myself, I hadn’t really talked about it or entered a process that made something happen […] but you become much more comfortable with yourself over time, and it’s sort of when you start the process [of medical treatment] that things improve a lot. (Jacob, 17 years)

Thus, the mere act of initiating medical treatment seems to create a beacon of hope and a sense of improved well-being. In summary, it seems that after starting medical treatment, participants feel closer to a sense of authenticity in how they experience themselves in relation to the external world, where they feel more recognized by others in terms of gender. This appears to be a contingent and ongoing process because the seemingly private experiences of bodily changes gain meaning in social life and enable new senses of self.

#### Sub-theme 3.2: family and friends are sources of support and meaning

To engage in the meaning-making work of connecting the internal sense of self with external expression while undergoing medical treatment, all participants emphasized that strong networks of social support have been pivotal. Friends, family, care providers, and peer support are important mediators of meaning and sources of social support. Having someone to share frustrations and vent with was mentioned as helpful:

I wouldn’t have been able to get to where I am if I hadn’t had my mom, because she’s a very patient person. She has taught me that you shouldn’t put your life on hold while waiting for something to happen. I’ve had that mindset when [for example] I find out there’s a waiting list for surgery; I was told it could take up to a year, and I’m not really a patient person. (Jacob, 17 years)

Jacob became tearful as he explained how important his mother has been in communicating hope, highlighting the significance of emotional support. Furthermore, it seems that a strong support network gave some participants confidence in the treatment process, which then made it possible for them to attend to other aspects of the transition: “I could relax in a way, I knew that the changes that were coming, were going to happen anyway, so I could think about other things” (Jacob, 17 years).

In addition to emotional support, social networks are important for exploring the wider context in which medical treatment takes place. Discussing the implications of trans politics on a daily basis with parents and family, for example, was mentioned as important for exploring the social meaning of gender-affirming care: “Dad brings it up sometimes if he sees that trans issues are debated on TV, and sometimes I see it, sometimes I don’t. It’s more thinking out loud [together], I guess” (William, 17 years). Furthermore, many participants have found support in peer groups with other young people who share similar experiences with gender-affirming care:

There are a surprising number of trans people in this country. There are, for example, separate Facebook groups and you can easily ask lots of questions there and get help. So, I did that quite a bit, especially in the beginning, when I was really unsure, “is this right when it comes to hormones? Does it have to happen so soon? Will it hurt that much to get that injection?.” It helped a lot in the beginning. And then I tried to help others who had questions as well. (Henrik, 22 years)

Thus, networks of social support can be used to find practical solutions to everyday challenges, as well as emotional help and meaning-making.

## Discussion

The current study explores the experiences of gender-affirming care in transgender and gender nonconforming (TGN) youth and young adults three years after they began medical treatment. The first major theme, *a pragmatic approach to decision-making regarding medical treatment*, indicates that participants continuously weigh the benefits against potential risks (sub-theme 1.1), and that these interventions are introduced step-by-step (sub-theme 1.2). As highlighted in the second major theme, participants have, to varying degrees, *changed their bodies through medical treatment*. Initially, these bodily changes are experienced as tangible (sub-theme 2.1), but over time they appear to contribute to an increased sense of masculinity (sub-theme 2.2). In this way, medical interventions seem to increase bodily alignment with gender identity in everyday life. However, this transition from tangible bodily changes to the psychological experience of increased masculinity occurs within a social framework where family, friends, or intimate partners are mediators of meaning. This leads to the third major theme, which suggests that *bodily changes need grounding in everyday life to become socially meaningful*. Furthermore, medical treatment helps connect the internal sense of self with the external world through social interaction and engagement with others (sub-theme 3.1). Family and friends are key sources of social support and mediators of meaning in this process (sub-theme 3.2). In the following sections, we first discuss the findings in light of empirical literature on the treatment experiences of individuals seeking medical treatment in gender-affirming care. Secondly, we address the importance of attending to psychosocial processes and support networks while implementing medical treatment in gender-affirming care in the section on clinical implications.

The results from the present study indicate that medical treatment aimed at changing the body to more closely align with gender identity can be beneficial for increasing appearance congruence, which refers to how transgender individuals perceive authenticity and comfort in their outward presentation (Chen et al., 2023; Sansfaçon et al., 2019). This is also in line with clinical guidelines for medical treatment in gender-affirming care (Coleman et al., 2022). The current study shows that, for binary transgender men, appearance congruence is particularly experienced as an increased sense of masculinity (sub-theme 2.2) and an improved ability to negotiate their internal sense of gender with their gender expression in relation to the external environment (sub-theme 3.1). In this, the present study supports recent outcome studies indicating that increases in appearance congruence are suited to improving bodily alignment with gender identity in TGN young people (Achille et al., 2020; Allen et al., 2019; Chen et al., 2023).

Furthermore, the findings from the present study shed light on the underlying psychosocial processes that make medical treatment effective in increasing appearance congruence. The study indicates that the implementation of medical treatment in gender-affirming care for TGN youth and young adults is a complex process, involving the integration of bodily changes into their everyday life to understand their social meaning. Additionally, the meaning attached to these bodily changes needs to be interpreted with the help of caregivers, family, and friends as mediators of meaning. In this way, the findings resonate with previous research that highlights the important role of an affirming social environment in enhancing mental health and well-being in TGN young people experiencing gender distress (Carlile, 2020; Kearns et al., 2021; Riggs et al., 2020). Furthermore, the results indicate that the social environment—typically consisting of parents, caregivers, and friends—facilitates the translation of bodily changes into socially meaningful experiences for transgender and gender-nonconforming (TGN) young people. Previous research indicates that medical interventions enable TGN young people to be perceived by others in accordance with their gender identity (Chen et al., 2023; Sansfaçon et al., 2019). The findings from the present study build on these insights, suggesting that the social environment’s role is not solely as a mirror of internal gender identity but also as a co-constructor of meaning around the implications of bodily changes.

The importance of other people, such as family and friends, and a supportive and non-judgmental social environment in interpreting bodily changes can also be understood through a queer reading. The subjective experience of inhabiting a body, referred to as embodiment, develops over time and is mediated by relationships with others. Thus, embodiment is invested in social and cultural meaning-making frameworks that render the body meaningful (Salamon, 2010). When applied to the findings from this study, the importance of social support in interpreting bodily changes suggests that the body is always represented and negotiated in the mind, in relation to others, and through the citation of social norms (Salamon, 2010). For the TGN youth and young adults who participated, bodily changes seem particularly important when negotiating intimacy, sexuality, and the meaning attached to masculinity and a sense of being male. In this way, the current study provides insight into some of the psychosocial processes embedded within medical treatment in gender-affirming care. Furthermore, increased appearance congruence following medical treatment does not happen automatically; it presupposes that young people actively engage in meaningful interactions to understand the social significance of bodily changes.

### Clinical implications

Overall, the results from the present study suggest that psychosocial support during medical transition is crucial because the beneficial effects of medical treatment in gender-affirming care, associated with increased appearance congruence, are related to the ability to explore the social meaning of gender and identity in everyday life. Furthermore, the results from the present study, especially the first major theme (a *pragmatic approach to making decisions regarding medical treatment*), indicate that parents and care providers seem to focus more on the importance of a thorough psychiatric assessment to ensure certainty about medical treatment and prevent future regret, while the third major theme (*grounding bodily changes in everyday life*) indicates that the participants themselves prioritize the need for emotional and practical support in their everyday lives. Perhaps this reflects, as indicated by sub-theme 3.1 (*medical treatment helps connect the inside with the outside*), a gap between what adults on the outside perceive to be support and the challenges faced by young people in their attempts to negotiate social environments to reconcile their inner sense of self in terms of gender with how they are perceived by others in relation to social norms. Based on the results from the present study, we suggest three insights for clinicians.

Firstly, to assist transgender and gender non-conforming (TGN) youth and young adults in connecting their internal gender identities with their external experiences, clinicians should focus on how gender is embedded in daily experiences. Care providers can help TGN young people explore their specific social dynamics and how their sense of gender is woven into their unique everyday matrix of relationships and social norms. The findings from the present study underscore a pragmatic approach to medical treatment, where decision-making involves weighing the benefits of various medical interventions against the potential risks in daily life. In a previous article discussing findings from the first wave of interviews with the same participants, we suggested that clinicians might benefit from using these everyday life topics as starting points to understand how gender and gender incongruence play out in daily life and how each medical treatment step attempts to address daily challenges (Jessen et al., 2021b). Based on the findings from the present study, clinicians might benefit from exploring topics such as body, intimacy, and identity exploration typically associated with the transition from adolescence to adulthood, in addition to the social meaning of masculinity.

Secondly, we suggest that medical treatments be introduced gradually. This means interventions should be initiated successively, allowing clients to continuously reflect on the social implications of bodily changes when making further treatment decisions. Such a step-by-step approach provides a meaning-making framework through which the clinical rationale, such as masculinizing the body to align with gender identity, is interpreted within the social context of these bodily changes, as indicated in sub-theme 2.2 (*bodily changes are anchoring a sense of masculinity*).

Thirdly, the clinical encounter should be seen as an affirmative social meeting where young people can explore their gender. The results from the present study indicate that the initiation of medical treatment represents a step in the right direction and serves as an affirmation of transgender identity by care providers and family. This finding aligns with qualitative studies suggesting that parental denial of hormonal treatment can be perceived as a lack of affirmation of self-understanding (Kearns et al., 2021; Riggs et al., 2020; Sansfaçon et al., 2019). Therefore, the clinical encounter is not a neutral space where clinicians merely observe; rather, it contributes to the evolving exploration of gender and identity among TGN youth and young adults. This insight is especially important given that thorough psychiatric and psychosocial assessments are recommended before making decisions regarding medical treatments in gender-affirming care for young people (Butler et al., 2018; Coleman et al., 2022). Caregivers should recognize that the clinical assessment process can provide insight into how gender is experienced by clients and integrated within a broader social context. By offering an affirming and non-judgmental space, clinicians can use these encounters to explore the social meanings of gender, both in everyday life and during clinical meetings.

### Limitations

There are five aspects of the present study that might pose limitations. Firstly, all participants are doing quite well in terms of employment and education, and their families are generally supportive. Furthermore, all participants are either in education or full-time work. This means we may not have captured the experiences of TGN youth and young adults who are more marginalized in terms of social support. Secondly, all participants were assigned female at birth, which excludes the experiences of TGN young people assigned male at birth. Thirdly, although the Method section notes that two participants are people of color, we chose to withhold specific details about their racial backgrounds in the Results section to ensure their anonymity. We recognize, however, that participants’ racial backgrounds can affect how they are perceived in terms of gender by others and, in turn, influence their experiences of gender and medical treatment. We therefore recommend that future research specifically explore the experiences of young transgender people of color to better understand the impact of racial background on gender-affirming care in Norway. Fourthly, all the participants live in Norway, a country with a strong welfare state and a population that generally reports positive attitudes toward trans and non-binary people. A final limitation is that all but one participant identifies as a binary transgender man. This is significant given the high prevalence of non-binary individuals among transgender youth and young adults (Twist & de Graaf, 2019), and emerging research suggesting that binary trans men might be more influenced by heteronormative scripts related to body and sexuality than their non-binary counterparts (Anzani et al., 2021). The gender experiences described in the present study may thus more closely reflect the specific challenges associated with having a binary identity as a transgender man and greater engagement with heteronormative scripts. Clinicians working with young non-binary trans people seeking medical treatment should be mindful of these potential differences.

### Future research

The present study indicates that the beneficial effects of medical treatments in achieving bodily alignment with gender identity are socially embedded. Future qualitative studies should therefore explore experiences with gender-affirming care at later stages in the treatment journey. Quantitative studies are needed to identify different developmental trajectories in the interplay between factors such as stigma, social support, and co-occurring mental health difficulties that contribute to distress in TGN young people. Ideally, such outcome studies should be combined with qualitative approaches that explore in-depth the interplay between different factors on an individual level in diverse young people receiving gender-affirming care.

## Conclusion

The present study indicates that medical treatment in gender-affirming care can be beneficial for TGN youth and young adults assigned female at birth in Norway, facilitating the integration of their internal sense of gender with their external environment. On an experiential level, bodily changes seem to anchor a sense of masculinity in daily life, offering new ways to experience and negotiate gender in everyday settings. Psychosocial support—typically provided by family, friends, and TGN peers—is crucial in this process, acting as a mediator of social meaning as tangible bodily changes are integrated into everyday life contexts. Clinicians should therefore aim to support TGN youth and young adults seeking medical treatment in aligning their internal sense of gender with the external world and in exploring the social meaning of bodily changes within their unique everyday life contexts. Furthermore, introducing medical treatment in a step-by-step manner could be beneficial, carefully weighing the risks against the benefits in daily life settings.

## Note

1 Cisgender refers to individuals who identify with their sex assigned at birth. The term has been developed as a binary alternative to transgender.
